# Not just the medial temporal lobe: Precuneus and posterior cingulate volumes relate to plasma biomarkers and cognition in a sub‐Saharan African cohort

**DOI:** 10.1002/alz.70768

**Published:** 2025-11-16

**Authors:** Maria Misiura, Alexandria Bartlett, Cadence Claar‐Pressley, Jennalyn Burnette, Chinkuli Munkombwe, Jean Ikanga

**Affiliations:** ^1^ Department of Psychology Georgia State University Atlanta Georgia USA; ^2^ Tri‐Institutional Center for Translational Research in Neuroimaging & Data Science Georgia State University Georgia Institute of Technology Emory University Atlanta Georgia USA; ^3^ Neuroscience Institute Georgia State University Atlanta Georgia USA; ^4^ Department of Anthropology Georgia State University Atlanta Georgia USA; ^5^ Women's Gender and Sexuality Studies Institute Georgia State University Atlanta Georgia USA; ^6^ Department of Rehabilitation Medicine Emory University School of Medicine Atlanta Georgia USA; ^7^ Department of Psychiatry School of Medicine University of Kinshasa and Catholic University of Congo Kinshasa Democratic Republic of the Congo

**Keywords:** Alzheimer's disease, neuroimaging, plasma biomarkers, posterior cingulate, precuneus, sub‐Saharan Africa

## Abstract

**INTRODUCTION:**

The precuneus and posterior cingulate—key regions in early Alzheimer's disease (AD)—undergo atrophy before clinical symptoms emerge. Limited research in sub‐Saharan Africa (SSA) has explored how blood‐based biomarkers relate to brain changes and cognition. This study examined these associations in older adults with dementia in Kinshasa, Democratic Republic of Congo (DRC).

**METHODS:**

A total of 117 adults (≥ 65 years; five dementia, and 58 controls) completed neuropsychological testing and blood draws, with 75 completing neuroimaging. Plasma amyloid‐beta (Aβ) 40, Aβ42, pTau181, pTau217, and neurofilament light chain (NfL) were analyzed using single molecule array (Simoa). Regional volumes were obtained via Freesurfer. Linear models assessed biomarker, volume, and cognitive associations.

**RESULTS:**

Lower Aβ42/40 and higher NfL levels were associated with reduced posterior cingulate and right precuneus volumes. Posterior cingulate atrophy correlated with poorer semantic fluency, memory, and executive function. Male sex and higher education were linked to larger volumes.

**CONCLUSIONS:**

Plasma Aβ42/40 and NfL are promising biomarkers of AD‐related brain atrophy in SSA. Results support region‐specific screening approaches in global dementia research.

**Highlights:**

- First biomarker‐neuroimaging study of dementia in sub‐Saharan Africa (SSA).
- Lower amyloid‐beta (Aβ) 42/40 linked to reduced posterior cingulate volumes.
- Higher plasma NfL associated with right precuneus and PCC atrophy.
- Posterior cingulate atrophy predicted memory and executive dysfunction.
- Study supports plasma biomarkers for AD screening in SSA settings.

## 1 BACKGROUND

The estimated number of adults living with Alzheimer's disease (AD) exceeds 55 million globally, and these estimates are projected to grow exponentially as the world population continues to age.^1^ AD pathology involves the accumulation of amyloid‐beta (Aβ) plaques and neurofibrillary tangles of tau proteins in the brain,^2^ which can be detected using a variety of fluid biomarkers. The Alzheimer's Association provides three broad categories of AD‐related fluid biomarkers: (1) core biomarkers, including Aβ42, pTau217, pTau181, pTau23, and MTBR‐tau243; (2) non‐specific biomarkers involved in AD pathophysiology, including neurofilament light chain (NfL) and glial fibrillary acidic protein (GFAP); and (3) biomarkers of non‐AD co‐pathology alpha synuclein and neuroimaging evidence of infarction.^3^ These biomarkers allow for early detection of AD neuropathology even before the emergence of observable cognitive or behavioral symptoms. While these biomarkers offer insight into the biochemistry of AD, neuroimaging provides critical information about how these pathological changes manifest in specific brain regions.

In structural and functional neuroimaging studies, the precuneus and posterior cingulate both exhibit atrophy and functional changes in individuals at risk for, or diagnosed with AD.^4^, ^5^ The precuneus and posterior cingulate are some of the first regions to be affected by the pathological changes in AD.^4^ These changes include the loss of gray matter, decreased cerebral perfusion, and reduced functional connectivity to other brain regions. Moreover, both the precuneus and posterior cingulate represent key components of the default mode network (DMN), a system of interconnected brain regions that is, more active during passive tasks. Although functional brain connectivity generally decreases with advancing age, those with AD demonstrate particular declines in the DMN beyond what would be expected with normal aging.^6^ As such, volumes in these two brain regions are regarded as initial sites of AD pathogenesis.

Precuneus and posterior cingulate volumes have shown several associations with blood‐based biomarkers of AD pathology. Results from systematic review suggest that lower Aβ42 and Aβ42/40 levels are correlated with lower hippocampal volume and increased Aβ deposition in the precuneus, that pTau217 and pTau181 are negatively correlated with gray matter volume in regions including the posterior cingulate gyrus, and NfL levels negatively correlate with gray and white matter structural integrity,^7^ starting within the precuneus.^8^ These blood‐based AD biomarkers and structural changes are also useful in predicting cognitive impairment. For instance, plasma pTau levels demonstrate adverse associations with cognition and volume loss on neuroimaging.^9^ Similarly, rising serum NfL levels have been associated with declining memory and language performance.^10^ Thus, combining structural imaging techniques with serum biomarkers and cognitive assessment data may facilitate more accurate and timely diagnosis of AD in vulnerable adults.

Despite consistent evidence demonstrating that persons of African descent are at higher risk for developing AD, relatively few studies have been conducted outside of Western cohorts.^11^ Thus, little is known about the associations with serum biomarkers of AD, structural brain regions, and cognitive performance in diverse populations. This is especially disappointing given that nearly 16% of the global population resides in sub‐Saharan Africa (SSA),^12^ and within that population, an estimated 2.13 million adults were living with dementia in 2015.^13^ Consequently, studies focused on this region are needed to better understand the pathogenic and clinical presentation of AD in this underexplored group. Further, studies incorporating blood‐based biomarkers may be especially useful in screening for AD pathology in areas such as SSA given their comparably lower cost and accessibility relative to established cerebrospinal fluid (CSF) measures that are more invasive, scarce, and costly to obtain.^14^, ^15^

Therefore, this project investigated associations between precuneus/posterior cingulate volumes with blood‐based AD biomarkers and cognitive performance in a sample of older adults in Kinshasa, the capital city of the Democratic Republic of Congo (DRC). Results from this study are among the first to describe how these plasma biomarkers relate to structural and functional brain changes in the SSA population, filling an important gap in the literature.

## 2 METHODS

Participants of this study are community‐dwellers from Kinshasa/DRC diagnosed with dementia and selected from a prevalence study of dementia. Study design details have been published previously. Briefly, participants were included if they were at least 65 years or older, had a family member or close friend to serve as an informant, and were fluent in French or Lingala. We excluded individuals who had a history of schizophrenia, neurological, or other medical conditions potentially affecting the central nervous system (CNS), yielding a sample of 1432 eligible participants.

A panel consisting of a neurologist, psychiatrist, and neuropsychologist reviewed screening tests, clinical interviews, and neurological examinations of 271 subjects, of whom 59 from 88 were confirmed with a diagnosis of dementia and 58 from 183 were considered healthy controls (HC). Among these 117 participants, 29 refused to provide blood samples, leaving 85 participants (75%) in whom plasma biomarkers were obtained (45 dementia and 40 HC) who were matched on age, education, and sex, of these participants 75 also had neuroimaging data (Table 1). Written informed consent was obtained prior to participants undergoing any study procedures. Participants were financially compensated for their time. The procedures were approved by the Ethics Committee/Institutional Review Boards of the University of Kinshasa and Emory University.

**TABLE 1 alz70768-tbl-0001:** Demographic table.

Parameter	All participants (*n* = 75)	Cognitively normal (*n* = 28)	Cognitively impaired (*n* = 47)
Age, years, mean (SD)	72.5 (8.1)	71.5 (8.7)	73.0 (7.7)
Gender (F/M)	35/40	18/10	25/22
Years of education mean (SD)	9.23 (5.35)	11.00 (4.97)^*^	8.17 (5.34)^*^
APOE4 genotype (*n* each)	4, 26, 21, 7	2, 16, 5	2, 10, 16, 7
Aβ42/40 (SD)	0.1027 (0.0095)	0.1080 (0.0081)^*^	0.0992 (0.0088)^*^
pTau181 (SD)	1.17 (1.12)	0.92 (0.69)	1.31 (1.29)
pTau217 (SD)	0.422 (0.453)	0.462 (0.504)	0.374 (0.391)

Abbreviations: Aβ, amyloid‐beta; APOE4, apolipoprotein E4; NfL, neurofilament light chain; pTau, phosphorylated tau; SD, standard deviation.

* Significantly different between normal and impaired groups.

**RESEARCH IN CONTEXT**

1. **Systematic review**: We conducted a literature review using PubMed and recent Alzheimer's conference abstracts. While blood‐based biomarkers and structural imaging markers are increasingly used in Alzheimer's disease (AD) research, few studies have examined these relationships in African cohorts. Prior work has focused predominantly on Western populations, limiting generalizability to underrepresented global regions. Relevant studies supporting biomarker–atrophy–cognition associations are cited.
2. **Interpretation**: Our findings demonstrate that lower Aβ42/40 and higher NfL are associated with posterior cingulate and right precuneus atrophy in older adults in the Democratic Republic of Congo. These atrophic patterns also relate to poorer semantic fluency, memory, and executive function. Results suggest early amyloid‐ and axon‐related neurodegeneration in a sub‐Saharan African cohort.
3. **Future directions**: Future work should validate these findings in larger, longitudinal SSA samples and incorporate tau PET or CSF biomarkers. Studies should also examine the role of vascular disease and sex differences in AD progression in African populations.

### 2.1 Procedures

Participants[Table alz70768-tbl-0001] underwent a comprehensive clinical evaluation, including cognitive testing, self‐report questionnaires, and standard psychiatric and neurological evaluations. Subjects were interviewed to obtain demographic, socioeconomic, and medical history and were subsequently administered cognitive testing with African Neuropsychological Battery (ANB) subtests. To establish neurological status in the absence of established diagnostic criteria for AD in SSA, we screened eligible participants using the Alzheimer's Questionnaire (AQ) and the Community Screening Instrument for Dementia (CSID).^16^ The AQ assesses activities of daily living and symptoms of AD in participants, and from this we derived three subscales: semantic or language fluency, memory performance, and executive function.^17^

### 2.2 Blood samples

Blood samples were drawn at the Medical Center of Kinshasa (CMK) blood laboratory by antecubital venipuncture into dipotassium ethylenediaminetetraacetic acid (K_2_ EDTA) tubes. Samples were centrifuged within 15 min at 1800 g at house temperature, and 5 mL of plasma was aliquoted into 0.5 mL polypropylene tubes and stored initially at −20°C for less than a week and stored in a −80°C freezer for longer term storage at a CMK laboratory. These aliquots were shipped frozen on dry ice to Emory University for storage and then to University of California San Francisco (UCSF) for measurements.

Plasma biomarker concentrations were measured using commercially available Neurology 4‐PLEX E (Aβ40, Aβ42, NfL; lot #503819), pTau181 (pTau181 v2; lot #503732) Quanterix kits on the Simoa HD‐X platform (Billerica, MA, USA) at UCSF. pTau217 was measured using the proprietary ALZpath pTau217 CARe Advantage kit (lot #MAB231122, ALZpath, Inc.) on the Simoa HD‐X platform (due to low sample availability).

For Aβ40, Aβ42, and NfL, all samples were measured above the lower limit of quantification (LLOQ) of 1.02 pg/mL, 0.378 pg/mL, 0.4 pg/mL, and 2.89 pg/mL, respectively. The average coefficient of variation (CV) for Aβ40, Aβ42, and NfL, were 6.0, 6.5, and 5, respectively. The average CV for pTau217, the LLOQ was 0.024 pg/mL and the average CV was 19.8%.

### 2.3 Neuroimaging

All subjects were imaged on a 1.5 Tesla MRI unit (Siemens, Magneton Sonata) scanner at HJ Hospitals in Kinshasa using the same standardized imaging acquisition protocol based on the Alzheimer's Disease Research Center (ADRC) protocol of Emory University. This consisted of sagittal volumetric T1‐weighted (MPRAGE), coronal T2‐weighted, and axial diffusion‐weighted, T2‐weighted, and T2‐FLAIR sequences. Typical acquisition parameters for the MPRAGE sequence were TR = 2200 ms, minimum full TE, TI = 1000 ms, flip angle = 8°, FOV = 25 cm, with a 192 × 184 acquisition matrix, yielding a voxel size of approximately 1.25 × 1.25 × 1.2 mm. The standard MPRAGE, with a shorter TE of 4 ms and a TI of 1000 ms, was used to achieve better contrast and image quality.[Fig alz70768-fig-0001]

Images were reviewed by a subspecialty certified neuroradiologist with 14 years of experience who was blinded to plasma biomarker levels. White matter hyperintensities were graded according to the Age‐Related White Matter Changes (ARWMC) scale. MPRAGE images were reoriented into the oblique coronal plane orthogonal to the principal axis of the hippocampal formation, and medial temporal lobe atrophy (MTLA) and entorhinal cortex atrophy (EriCa) scores were assessed. Finally, the presence or absence of any additional abnormalities was noted, and patients were excluded if neuroimaging evidence indicated an etiology other than probable AD (e.g., presence of a brain tumor).

### 2.4 Quantitative volumetric analysis using Freesurfer

The 3D T1 images were segmented using Freesurfer ^18^(v6, MGH, MA, USA), which includes a full processing stream for MR imaging data that involves skull‐stripping, bias field correction, registration, and anatomical segmentation as well as cortical surface reconstruction, registration, and parcellation. Regional brain volume for both cortical and subcortical brain regions were calculated. For brain volumes, we calculated each volume as a ratio of intracranial volume.[Fig alz70768-fig-0002]

### 2.5 Statistical analyses

We constructed linear regression models to compare precuneus and posterior cingulate (right and left hemispheres) volumes as outcome variables. Plasma Aβ42/40, pTau181, pTau217, and three AQ subsets (semantic function, executive function, and memory) were our dependent variables. Models were adjusted for age, gender, and years of education (for models including cognitive variables). We corrected for multiple comparison's using the Bonferroni family‐wise error rate (FWER) correction method.

## 3 RESULTS

### 3.1 Dementia status

Among the 75 participants[Fig alz70768-fig-0003] with neuroimaging data, 28 were cognitively normal and 47 were diagnosed with dementia. The mean age did not significantly differ between individuals with and without dementia (71.5 vs. 73.0 years; *t* [53.94] = –0.76, *p* = 0.45; Welch's *t*‐test). However, participants with dementia had significantly fewer years of education compared to controls (mean = 8.17 [SD = 5.34] vs. 11.00 [SD = 4.97] years; *t* [62.72] = 2.34, *p* = 0.022; Welch's *t*‐test). There were also significant differences in plasma biomarker levels: individuals with dementia exhibited lower Aβ42/40 ratios (0.0992 vs. 0.1080; *t* [63.22] = 4.45, *p* < 0.001; Welch's *t*‐test), suggesting higher amyloid burden. The groups did not differ significantly in gender distribution (female/male: 10/18 in controls vs. 25/22 in the dementia group; chi‐squared test not shown). Apolipoprotein E (APOE) genotype distribution varied across groups but was not tested for significance due to small subgroup counts.[Fig alz70768-fig-0004], [Fig alz70768-fig-0005]

**FIGURE 1 alz70768-fig-0001:**
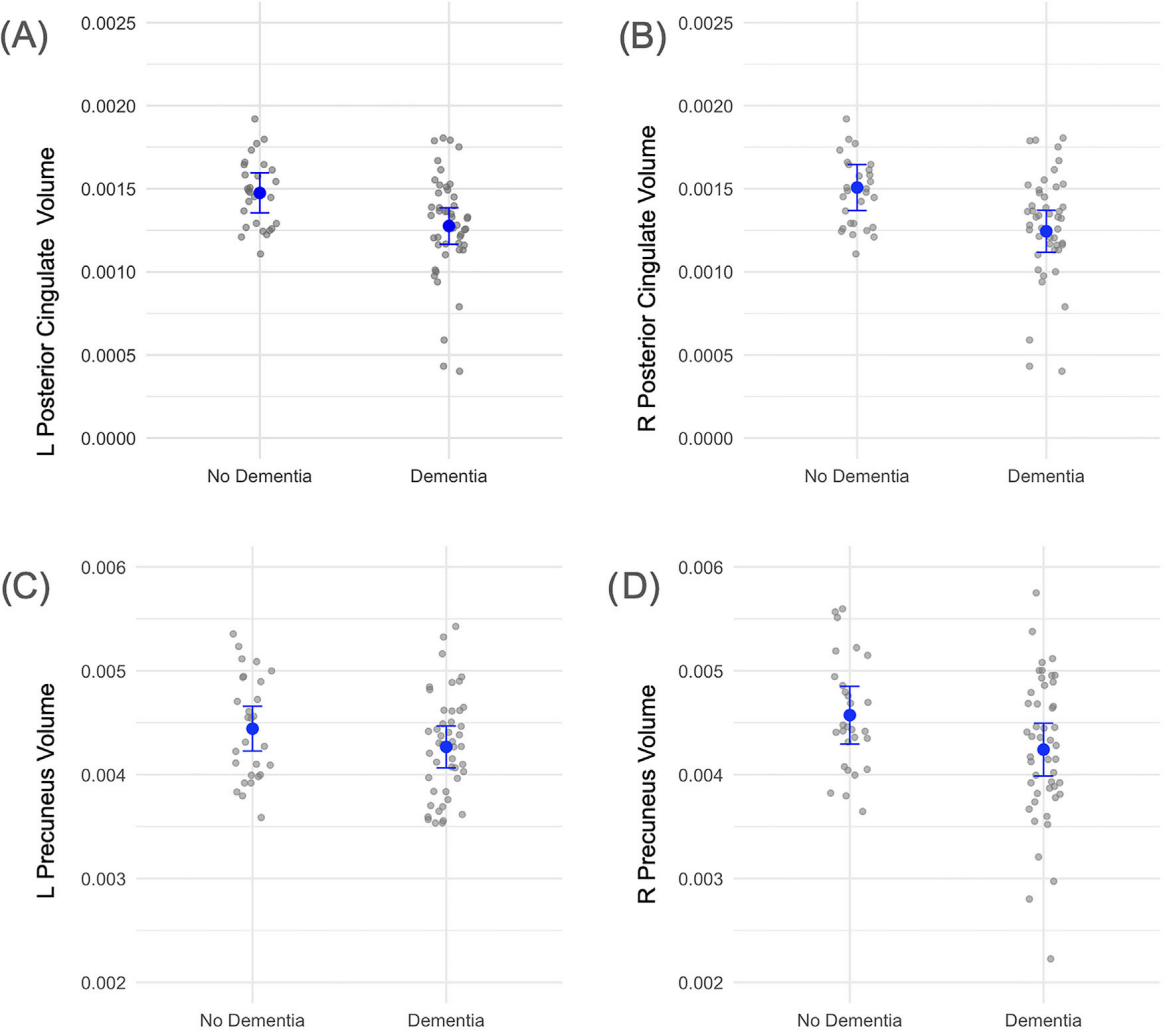
Relation between dementia status and regional brain volumes. (A) Left hemisphere posterior cingulate volume by dementia status (*β* = –1.77 × 10^−4^, *p* = 0.007). (B) Right hemisphere posterior cingulate volume by dementia status (*β* = –2.39 × 10^−4^, *p* = 0.0019). (C) Left hemisphere precuneus volumes by dementia status (*β* = –2.11 × 10^−4^, *p* = 0.07). (D) Right hemisphere precuneus volumes by dementia status (*β* = –2.81 × 10^−4^, *p* = 0.06). Brain volumes were normalized to intracranial volume. Each point represents an individual participant. Blue lines indicate estimated marginal means derived from linear regression models adjusting for age and gender. Error bars represent 95% confidence intervals around the adjusted means. The dispersion of gray points reflects the distribution of individual‐level volume data.

After adjusting for age and gender, neurological status was a significant predictor of posterior cingulate volume. Individuals with dementia exhibited lower volumes in both hemispheres, with significant associations observed in the left (*β* = –1.77e‐04, *p* = 0.007) (Figure 1A) and right posterior cingulate (*β* = –2.39e‐04, *p* = 0.0019) (Figure 1B). The effect was stronger in the right hemisphere. In contrast, precuneus volumes were not significantly related to neurological status (Figure 1C), although the right hemisphere showed a marginal trend toward reduced volume among individuals with dementia (β = –2.81e‐04, *p* = 0.060) (Figure 1D).

### 3.2 Plasma Aβ42/40

**FIGURE 2 alz70768-fig-0002:**
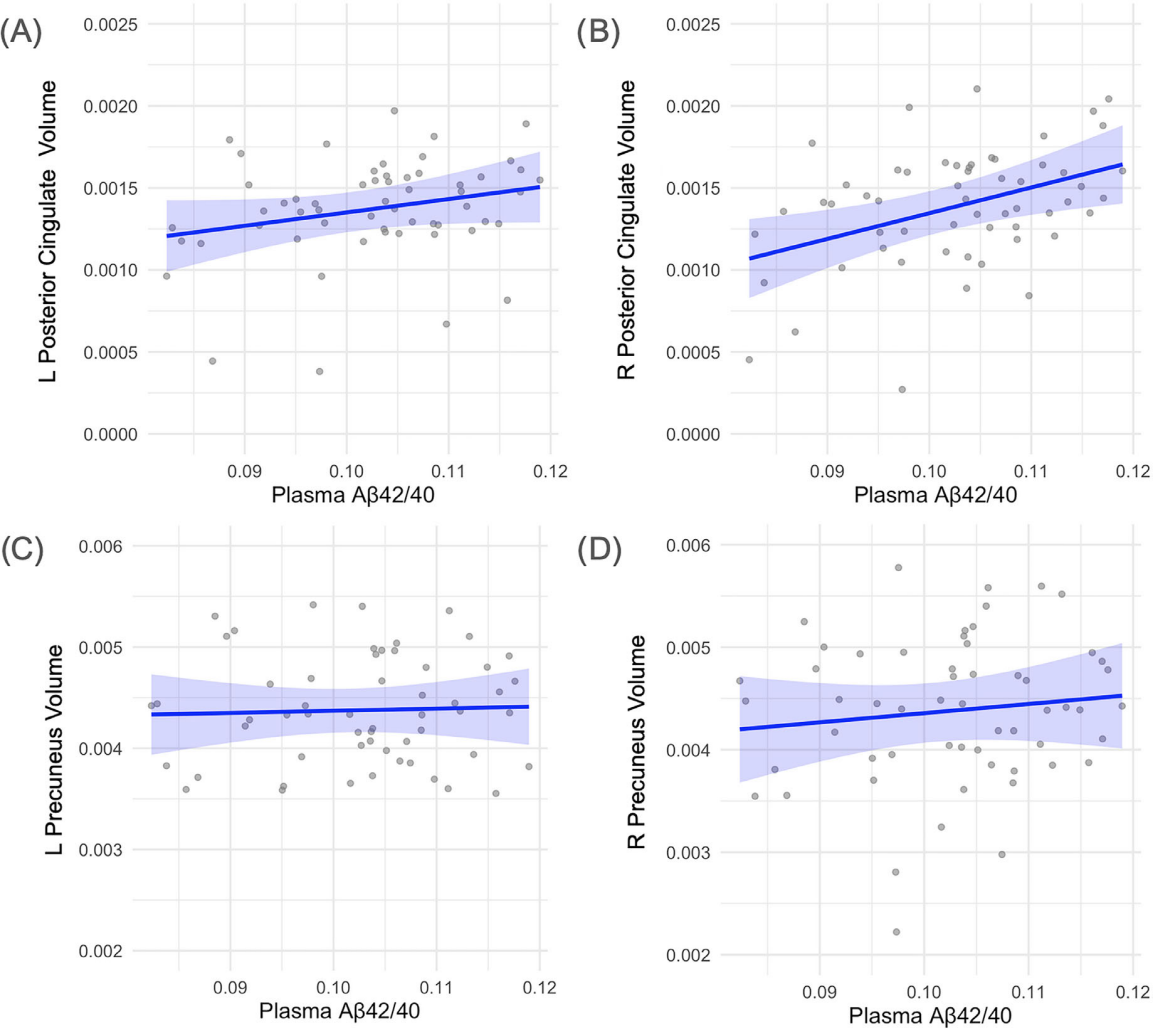
Relation between Aβ42/40 ratios and regional brain volumes. (A) Left hemisphere posterior cingulate volume by Aβ42/40 ratio (*β* = 0.0024, *p* = 0.044). (B) Right hemisphere posterior cingulate volume by Aβ42/40 ratio (*β* = 0.0021, *p* = 0.131). (C) Left hemisphere precuneus volume by Aβ42/40 ratio (*β* = 0.0021, *p* = 0.810). (D) Right hemisphere precuneus volume by Aβ42/40 ratio (*β* = 0.0089, *p* = 0.447). Brain volumes were normalized to intracranial volume. Each point represents an individual participant. Blue lines indicate estimated marginal means derived from linear regression models adjusting for age and gender. Shaded regions represent 95% confidence intervals around the fitted regression lines. The dispersion of gray points reflects the distribution of individual‐level volume data. Aβ, amyloid‐beta.

Lower Aβ42/40 ratios were associated with reduced posterior cingulate volumes, reaching significance in the left hemisphere (*β* = 0.0024, *p* = 0.044) and showing a non‐significant trend in the right (*β* = 0.0021, *p* = 0.131). No significant associations were observed in the precuneus for either hemisphere (left: *β* = 0.0021, *p* = 0.810; right: *β* = 0.0089, *p* = 0.447) (results are displayed in Figure 2).

### 3.3 Plasma pTau181 and pTau217

Neither pTau181 nor pTau217 were significantly associated with volumes in the precuneus or posterior cingulate, regardless of hemisphere. All associations were weak and non‐significant (e.g., left posterior cingulate: pTau181 *β* = –0.000024, *p* = 0.294; pTau217 *β* = 0.000055, *p* = 0.561) (results in Figures S1 and S2).

### 3.4 NfL

**FIGURE 3 alz70768-fig-0003:**
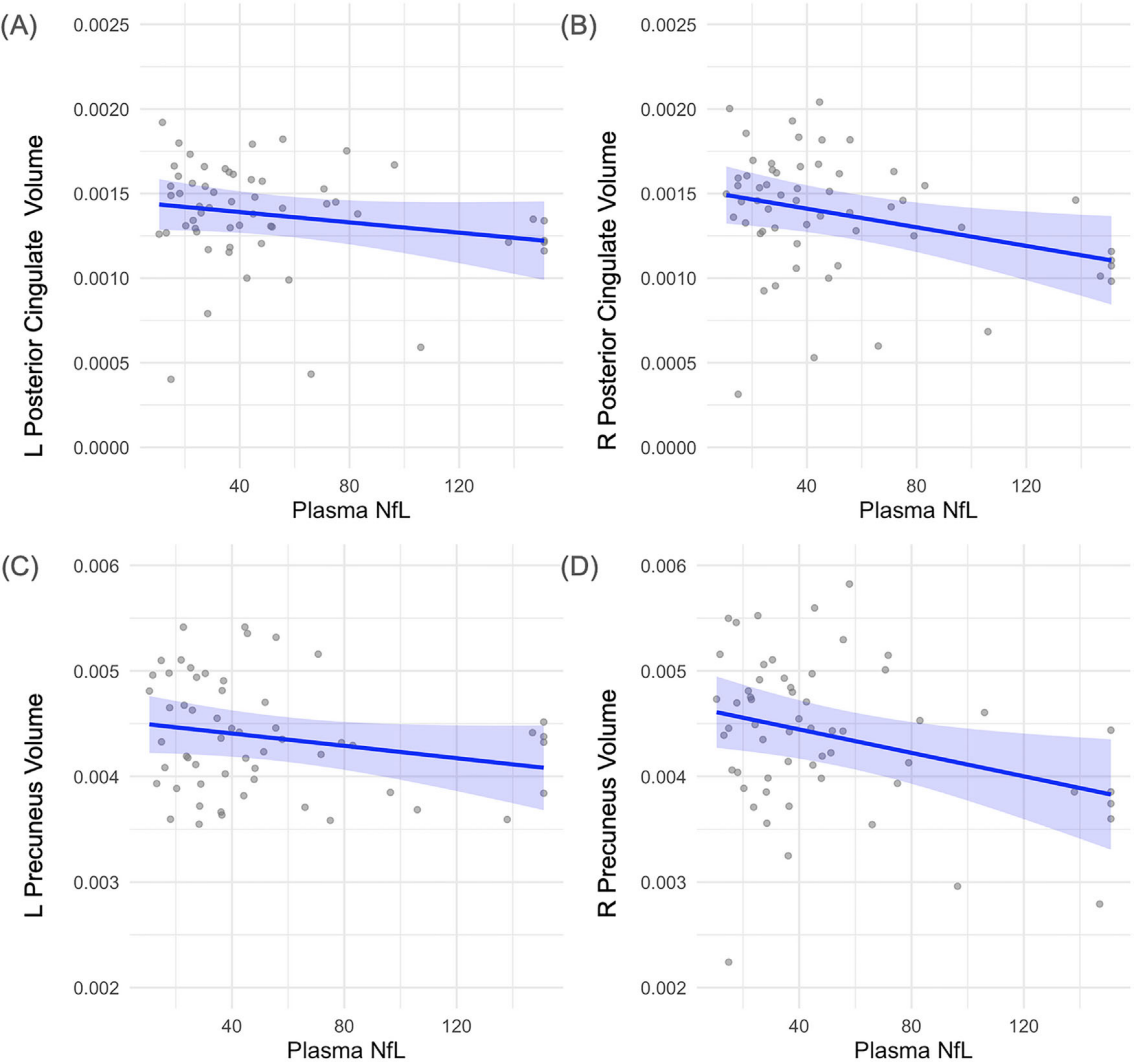
Relation between plasma NfL levels and regional brain volumes. (A) Left hemisphere posterior cingulate volume (*β* = –1.52 × 10^−^⁶, p = 0.147). (B) Right hemisphere posterior cingulate volume (*β* = –2.75 × 10^−^⁶, *p* = 0.022). (C) Left hemisphere precuneus volume (*β* = –2.92 × 10^−^⁶, *p* = 0.111). D) Right hemisphere precuneus volume (*β* = –5.55 × 10^−^⁶, *p* = 0.021). Brain volumes were normalized to intracranial volume. Each point represents an individual participant. Blue lines indicate estimated marginal means derived from linear regression models adjusting for age and gender. Shaded regions represent 95% confidence intervals around the fitted regression lines. The dispersion of gray points reflects the distribution of individual‐level volume data. NfL, neurofilament light chain.

Higher plasma NfL levels were significantly associated with lower volumes in the right precuneus (*β* = –5.55e‐06, *p* = 0.021) and right posterior cingulate (*β* = –2.75e‐06, *p* = 0.022), indicating a relationship between axonal damage and regional atrophy. Associations in the left hemisphere for both regions were non‐significant, though trends were directionally consistent (left precuneus: *β* = –2.92e‐06, *p* = 0.111; left posterior cingulate: *β* = –1.52e‐06, *p* = 0.147) (results displayed in Figure 3).

### 3.5 Cognition

**FIGURE 4 alz70768-fig-0004:**
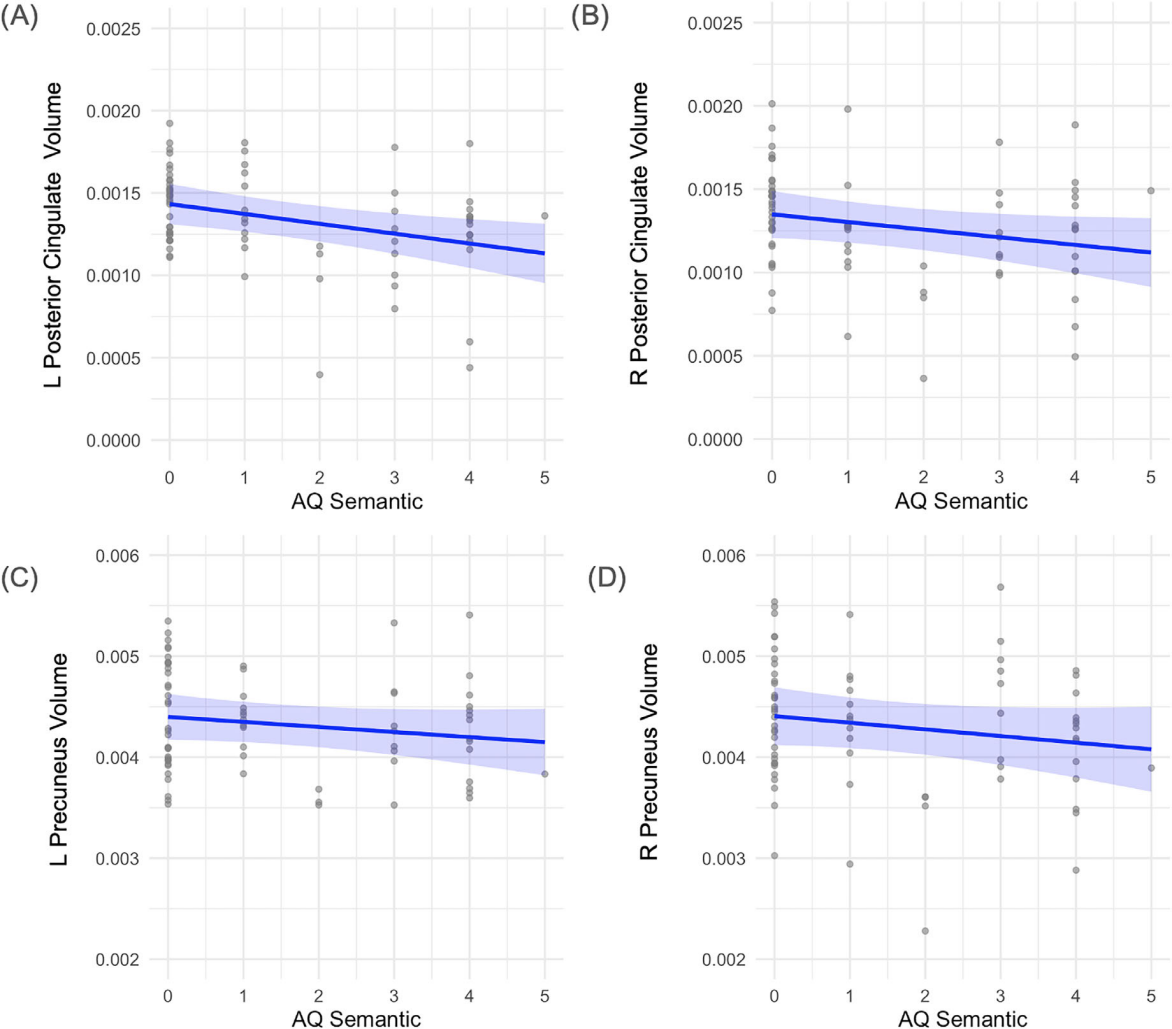
Relation between semantic fluency and regional brain volumes. (A) Left hemisphere posterior cingulate volume (*β* = –6.0 × 10^−^⁵, *p* = 0.005). B) Right hemisphere posterior cingulate volume (*β* = –4.6 × 10^−^⁵, *p* = 0.059). (C) Left hemisphere precuneus volume (no significant association, data shown for completeness). (D) Right hemisphere precuneus volume (no significant association, data shown for completeness). Brain volumes were normalized to intracranial volume. Each point represents an individual participant. Blue lines indicate estimated marginal means derived from linear regression models adjusting for age and gender. Shaded regions represent 95% confidence intervals around the fitted regression lines. The dispersion of gray points reflects the distribution of individual‐level volume.

**FIGURE 5 alz70768-fig-0005:**
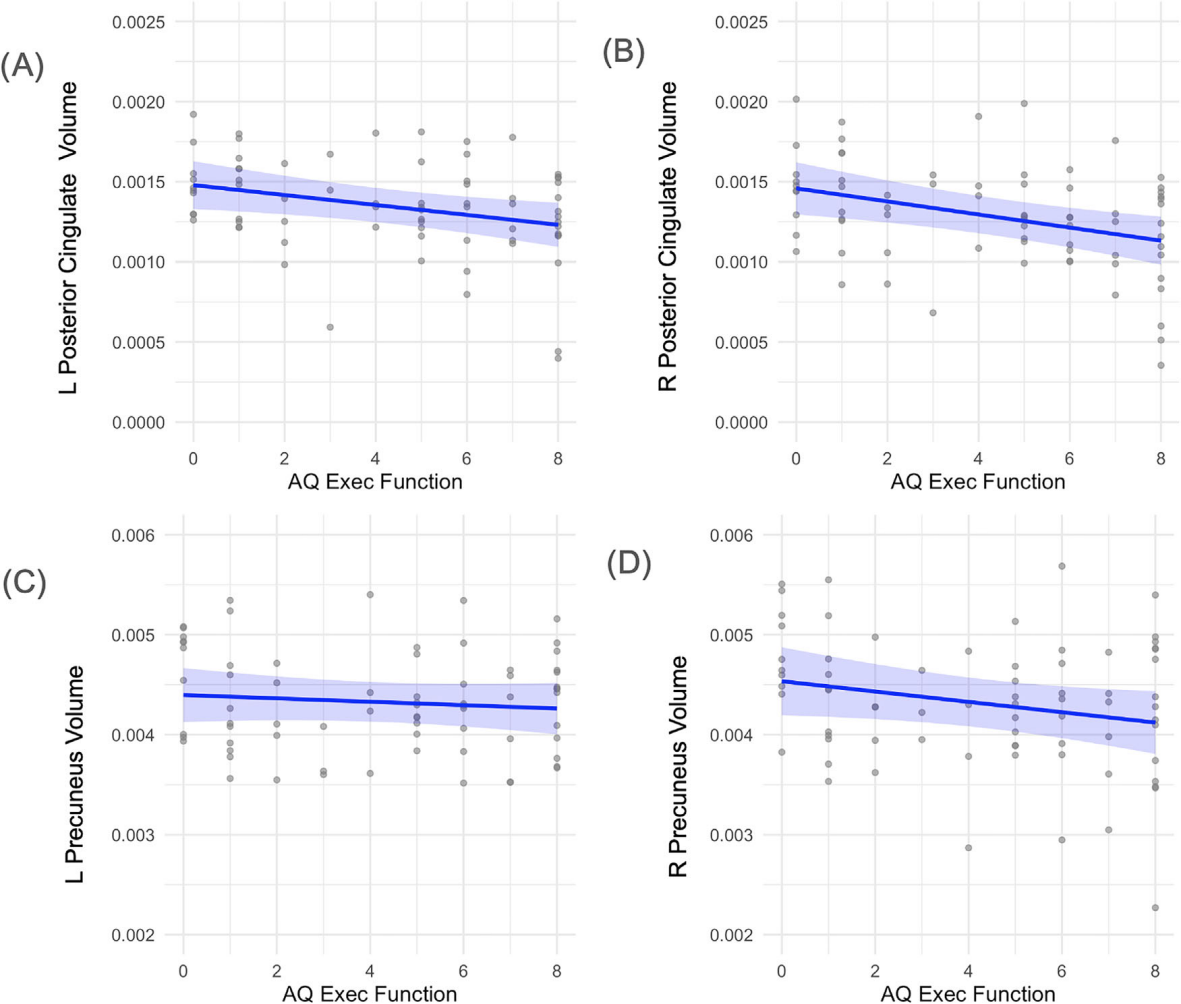
Relation between executive function and regional brain volumes. (A) Left hemisphere posterior cingulate volume (*β* = –3.1 × 10^−^⁵, *p* = 0.012). (B) Right hemisphere posterior cingulate volume (*β* = –4.1 × 10^−^⁵, *p* = 0.003). (C) Left hemisphere precuneus volume (no significant association, data shown for completeness). (D) Right hemisphere precuneus volume (no significant association, data shown for completeness). Brain volumes were normalized to intracranial volume. Blue lines indicate estimated marginal means derived from linear regression models adjusting for age and gender. Shaded regions represent 95% confidence intervals around the fitted regression lines. The dispersion of gray points reflects the distribution of individual‐level volume data.

**FIGURE 6:**
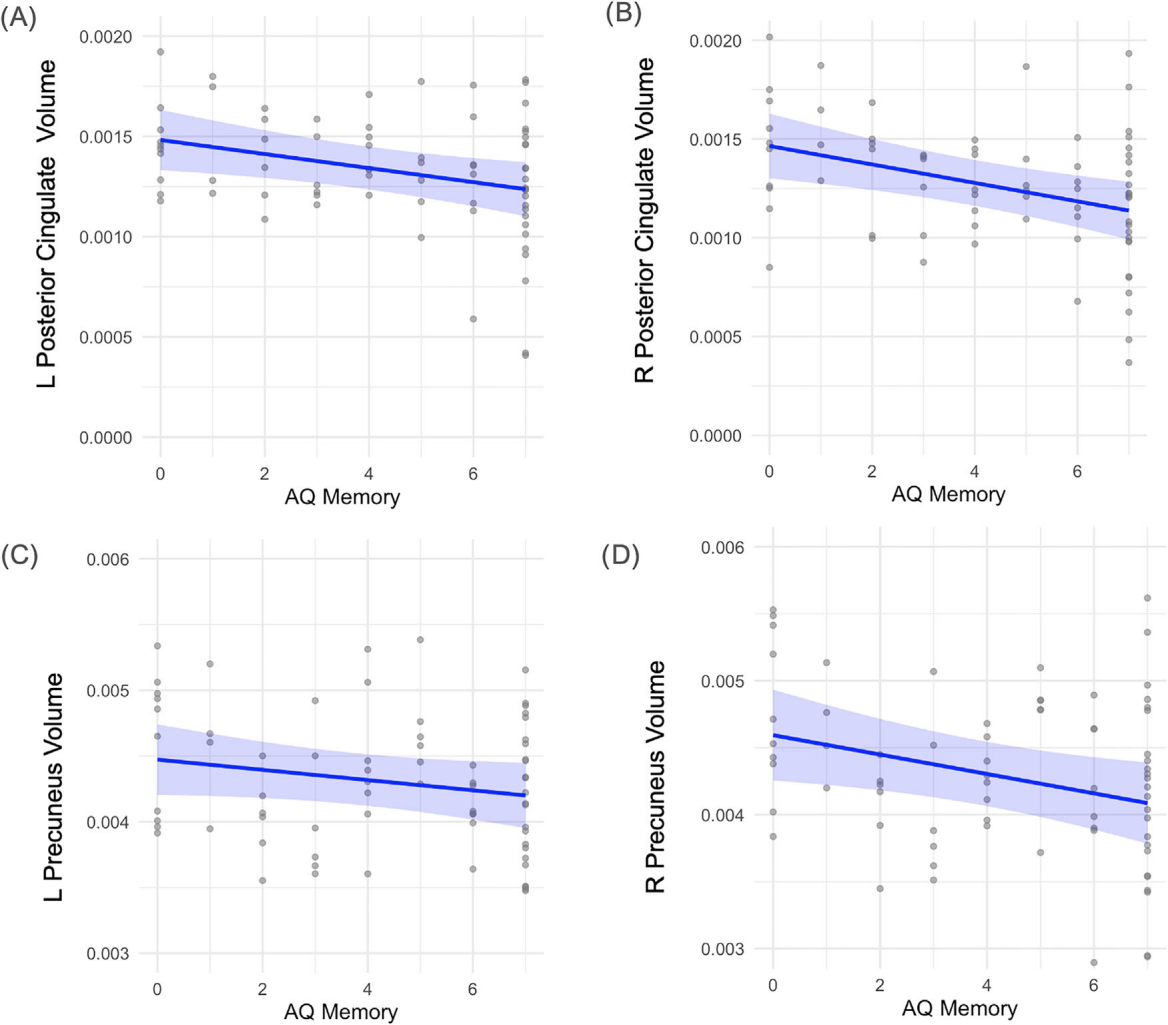
Relation between memory performance and regional brain volumes. (A) Left hemisphere posterior cingulate volume (*β* = –2.1 × 10^−^⁵, *p* = 0.005). (B) Right hemisphere posterior cingulate volume (*β* = –2.6 × 10^−^⁵, *p* = 0.001). (C) Left hemisphere precuneus volume (no significant association, data shown for completeness). (D) Right hemisphere precuneus volume (*β* = –4.3 × 10^−^⁵, *p* = 0.006). Brain volumes were normalized to intracranial volume Each point represents an individual participant. Blue lines indicate estimated marginal means derived from linear regression models adjusting for age and gender. Shaded regions represent 95% confidence intervals around the fitted regression lines. The dispersion of gray points reflects the distribution of individual‐level volume data.

Semantic fluency was significantly associated with left posterior cingulate volume (*β* = –0.000060, *p* = 0.005) and showed a marginal relationship in the right hemisphere (*β* = –0.000046, *p* = 0.059). No significant associations were observed with precuneus volume (results displayed in Figure 4). Executive function scores were also related to posterior cingulate structure, with significant associations in both hemispheres (left: *β* = –0.000031, *p* = 0.012; right: *β* = –0.000041, *p* = 0.003), but not in the precuneus (results displayed in Figure 5). Memory performance was significantly linked to volume reductions in the right precuneus (*β* = –0.000043, *p* = 0.006) and in both hemispheres of the posterior cingulate (left: *β* = –0.000021, *p* = 0.005; right: *β* = –0.000026, *p* = 0.001). No significant relationship was found between memory and left precuneus volume. (Results displayed in Figure 6). Additionally, male sex and higher education were associated with greater right posterior cingulate volume (gender: *β* = 0.00032, *p* = 0.001; education: *β* = 0.000020, *p* = 0.059).

Table 2 shows a summary table of results for all AD biomarkers across the left and right hemispheres of the precuneus and posterior cingulate.

**TABLE 2:** Summary table of blood biomarker and cognition results by left and right precuneus and posterior cingulate.

AD biomarker	Region	Estimate	*p*‐Value	95% CI
Aβ42/40	Left precuneus	0.0021	0.81	−0.23–0.31
	Right precuneus	0.0089	0.447	−0.16–0.36
	Left posterior cingulate	0.0024	**0.0440**	0.01–0.50
	Right posterior cingulate	0.0021	0.131	−0.06–0.45
pTau181	Left precuneus	−0.000053	0.454	−0.38–0.14
	Right precuneus	−0.000069	0.478	−0.40–0.11
	Left posterior cingulate	−0.000024	0.294	−0.40–0.11
	Right posterior cingulate	−0.000015	0.574	−0.36–0.16
pTau217	Left precuneus	−0.00015	0.422	−0.42–0.17
	Right precuneus	0.000012	0.96	−0.31–0.28
	Left posterior cingulate	0.000055	0.561	−0.24–0.35
	Right posterior cingulate	0.000061	0.604	0.30–0.30
NfL	Left precuneus	−0.000003	0.111	−0.44–0.05
	Right precuneus	−0.000006	**0.0206**	−0.53–0.07
	Left posterior cingulate	−0.000002	0.147	−0.44–0.05
	Right posterior cingulate	−0.000003	**0.0220**	−0.53–0.07
AQ executive	Left precuneus	−0.000017	0.439	−0.33–0.13
	Right precuneus	−0.000041	0.12	−0.43–0.00
	Left posterior cingulate	−0.000031	**0.0120**	−0.49–0.06
	Right posterior cingulate	−0.000041	**0.0030**	−0.53–0.12
AQ memory	Left precuneus	−0.000024	0.0712	−0.43–0.01
	Right precuneus	−0.000043	**0.0060**	−0.54–0.13
	Left posterior cingulate	−0.000021	**0.0050**	−0.51–0.09
	Right posterior cingulate	−0.000026	**0.0010**	−0.53–0.11
AQ semantic	Left precuneus	−0.00005	0.187	−0.39–0.06
	Right precuneus	−0.000063	0.166	−0.38–0.07
	Left posterior cingulate	−0.000060	**0.0050**	−0.51–0.09
	Right posterior cingulate	−0.000046	0.059	−0.43–0.01

Abbreviations: Aβ, amyloid‐beta; AD, Alzheimer's disease; AQ, Alzheimer's Questionnaire; CI, confidence interval; NfL, neurofilament light chain; pTau, phosphorylated tau.

Significance of bold *p*‐values < 0.05.

## 4 DISCUSSION

This study examined the relationships between AD‐related biomarkers, cognitive performance, and volumetrics in key regions associated with early AD pathology. After adjusting for age, gender, and years of education, we found that lower Aβ42/40 was significantly associated with reduced left posterior cingulate volume, whereas pTau181 and pTau217 did not show significant associations with precuneus or posterior cingulate volumes. Cognitive performance, measured through the posterior cingulate subdomains, showed specific associations with brain structure. Importantly, gender differences were observed, with males exhibiting significantly greater right posterior cingulate volume. Years of education showed marginal associations with right posterior cingulate volume, suggesting a potential cognitive reserve effect.

Our findings support previous research indicating associations between amyloid pathology (Aβ42/40) and brain volumes, particularly in the posterior cingulate cortex, a region known for its vulnerability in preclinical AD. However, in contrast with previous works, we did not observe significant relationships between pTau biomarkers (pTau181, pTau217) and brain volumes, instead observing similar pTau levels between those with and without dementia. For example, some PET studies have identified increased tau deposition in the precuneus and posterior cingulate,^19^, ^20^ while others demonstrated lower volumes at higher levels of plasma pTau181.^21^, ^22^ A potential reason for the lack of relationship in our cohort could be that lower tau levels have been identified in African individuals compared to other ethno‐racial groups.^23^ Further, previous analyses in this cohort revealed many exhibited mixed vascular pathology instead of “pure” AD^24^; thus, a greater vascular disease or damage burden could produce symptoms of dementia with lower tau.^24^, ^25^

The relationship between lower Aβ42/40 and posterior cingulate atrophy supports the hypothesis that amyloid accumulation disrupts structural integrity before detectable tau‐mediated changes occur. This suggests that amyloid burden may be an early trigger of neurodegeneration, making it a crucial target for early intervention. Additionally, the link between posterior cingulate atrophy and poorer semantic fluency, executive function, and memory suggests that structural decline in this region contributes to early cognitive dysfunction. Given that executive function was associated with both left and right posterior cingulate atrophy, this may reflect widespread disruption of connectivity networks crucial for attentional control and higher‐order cognition, which aligns with prior research linking this region to episodic memory and executive attention.^26^ These findings have clinical implications for biomarker‐driven screening tools, particularly in identifying individuals at risk for AD before significant cognitive impairment occurs. The observed sex effect, with males exhibiting greater right posterior cingulate volume, is consistent with studies showing sex differences in neurodegeneration,^27^ potentially influenced by hormonal,^28^ genetic, or vascular factors.

Our findings align with prior research in African American cohorts, particularly studies led by Misiura et al., which have highlighted the precuneus as a critical hub in early AD pathology.^29^, ^30^ In these studies, CSF Aβ42 levels were found to correlate with precuneus functional connectivity in Black Americans, suggesting that amyloid accumulation in this region may disrupt neural networks essential for cognitive function. This relationship was not observed in non‐Hispanic White individuals, indicating potential racial differences in AD biomarker presentation.

In line with prior studies, our findings suggest that elevated plasma NfL is associated with regional brain atrophy, particularly in the right hemisphere. Specifically, higher NfL levels were significantly related to lower volumes in the right precuneus and right posterior cingulate, two regions critically involved in memory and attentional networks and known to be vulnerable in early AD. The right hemisphere also showed stronger cognitive associations, suggesting lateralized vulnerability in AD progression. These results are consistent with previous research demonstrating that NfL reflects axonal damage and correlates with neurodegeneration across multiple modalities, including structural MRI and FDG‐PET.^31^, ^32^ For example, studies in both preclinical and clinically diagnosed AD populations have shown that plasma NfL is linked to cortical thinning and hypometabolism in DMN hubs, including the precuneus and posterior cingulate cortex.^33^ Our findings extend this work to an African cohort, where biomarker‐based studies remain scarce, and support the cross‐cultural generalizability of NfL as a marker of neuroaxonal injury. Previous work identified patterns of right lateralization such that right hemispheres tend to exhibit more atrophy earlier, which could explain our stronger rightward findings.^34^ The lateralized pattern observed may reflect differential vulnerability or compensatory mechanisms, which have been reported in other NfL studies but remain poorly understood.^35^ Further longitudinal research is needed to determine whether NfL predicts progressive regional atrophy or cognitive decline in this population.

A key strength of this study is its multimodal approach, combining plasma biomarkers, neuroimaging, and cognitive assessments to explore early AD pathology. Additionally, the inclusion of sex‐ and education‐related factors provides insights into individual differences in neurodegeneration. Future research should focus on longitudinal studies to track the progression of Aβ42/40, pTau biomarkers, and structural atrophy over time. This would clarify whether tau‐mediated neurodegeneration emerges later, following an amyloid‐driven phase of cortical thinning. Further, examining functional connectivity changes in the precuneus and posterior cingulate may provide more insights into early disruptions in AD‐related networks.

Our findings should be considered in the context of their limitations. The cross‐sectional nature of the study prevents us from determining causal relationships between biomarkers, brain atrophy, and cognitive performance. Additionally, the relatively small sample size may have reduced our ability to detect subtle biomarker associations, particularly with pTau181 and pTau217. We also did not stratify analyses by dementia diagnosis based on the goals of our study. Future studies with longitudinal follow‐up and analyses by dementia diagnosis will be essential to determine whether tau‐mediated neurodegeneration emerges at later disease stages. Because we identified volumetric differences between men and women, future studies should consider stratifying analyses by sex to investigate its role in these brain biomarker relationships, further improving our understanding of sex hormones in the diagnostic utility of blood biomarkers and neuroimaging to track disease progression. Other limitations of this study include potential biases and measurement error. Confounding may have also been present due to the mismeasurement of confounding variables. Future studies should use higher‐resolution MRI if available, standardize measurement protocols, blind data collectors to the outcomes and hypotheses, ensure proper consideration of confounding variables based on previous literature, and improve pre‐analytic quality to ensure valid plasma biomarkers.

As with our previous studies,^14^, ^24^ this study included only HC and participants with suspected dementia. Future studies should analyze all four groups (HC, mild cognitive impairment [MCI], subjective memory complaints, and dementia) to provide a more comprehensive understanding of the disease spectrum and the continuum of cognitive decline. In this study, we used plasma biomarkers due to the limited availability of confirmatory diagnostics, such as CSF or amyloid PET imaging biomarkers, to validate AD pathology. Future studies should include CSF analysis to confirm AD diagnosis and strengthen the validity of associations between plasma biomarkers and the precuneus.

In summary, our findings suggest Aβ42/40 is associated with posterior cingulate atrophy, whereas pTau biomarkers show no significant relationships with brain volume in this sub‐Saharan African sample. Cognitive performance—particularly semantic fluency, executive function, and memory—was linked to posterior cingulate atrophy, with the right hemisphere showing the strongest associations. Gender differences were also observed, highlighting the need for sex‐specific analyses in AD research.

These results underscore the importance of integrating multimodal biomarkers for early AD detection and suggest that amyloid‐driven structural changes may precede tau‐mediated neurodegeneration. Future research should focus on longitudinal tracking of biomarker progression, functional network disruptions, and individualized risk prediction models to further elucidate the early mechanisms of AD.

## AUTHOR CONTRIBUTIONS

Primary author of manuscript responsible for much of the content, analytical design, and communication between authors: A. Bartlett, Assisted with the writing of the introduction, discussion, and literature review; C. Claar‐Pressley, Assisted with the statistical analyses, table, and figure generation; J. Burnette, Assisted with literature search, manuscript formatting, and writing the abstract; C. Munkombwe, Assisted with preliminary analyses, ongoing collaboration, project inception, and manuscript preparation. J. Ikanga, Recruited and collected participants and serves as principal investigator for this project.

## CONSENT

All participants gave their consent for their de‐identified data to be used for the analyses for this publication.

## CONFLICT OF INTEREST STATEMENT

Maria Misiura, Alexandria Bartlett, Cadence Claar‐Pressley, Jennalyn Burnette, Chinkuli Munkombwe, and Jean Ikanga have nothing to disclose. Author disclosures are available in the Supporting Information.

## Supporting information



Supporting Information

Supporting Information

## Data Availability

The datasets during and/or analyzed during the current study available from the corresponding author on reasonable request. This study was approved by the Emory IRB and participants consented to share their data. All procedures were approved by the Ethics Committee/Institutional Review Boards of the University of Kinshasa.
