# Supplementary material for: Not just the medial temporal lobe: Precuneus and posterior cingulate volumes relate to plasma biomarkers and cognition in a sub‐Saharan African cohort
Source: Alzheimers Dement. 2025 Nov 16;21(11):e70768. doi: 10.1002/alz.70768 (PMC12620077; doi:10.1002/alz.70768)
Supplement: Supplementary file 1 — Supporting Information [file ALZ-21-e70768-s001.docx]

**
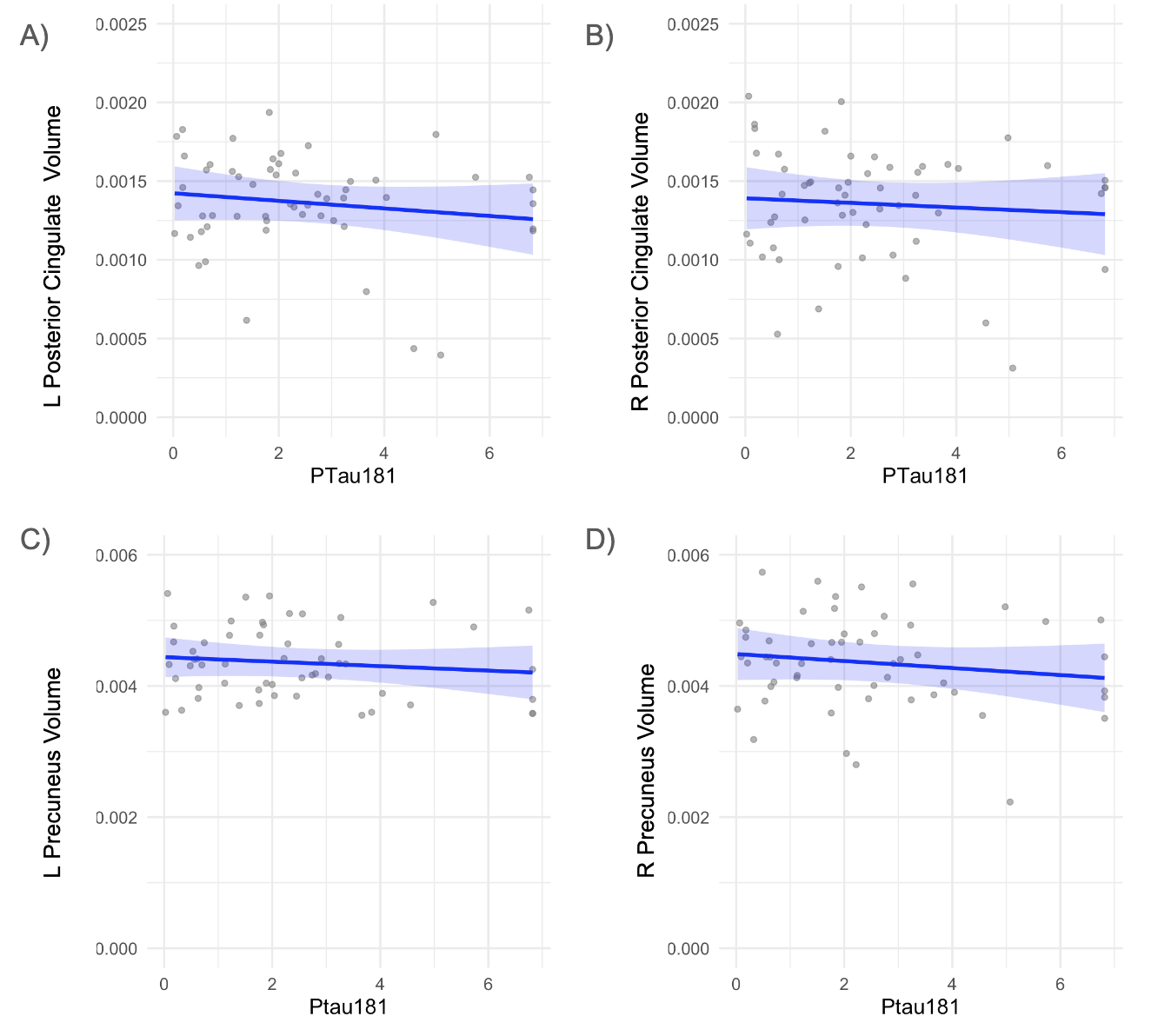
 Supplementary Figure 1.** Associations between plasma **pTau181** and regional brain volumes.Panels display linear regression models relating plasma pTau181 to (A) left precuneus, (B) right precuneus, (C) left posterior cingulate, and (D) right posterior cingulate volumes. None of the associations reached statistical significance (left precuneus: β = –5.30×10⁻⁵, p = 0.454; right precuneus: β = –6.90×10⁻⁵, p = 0.478; left posterior cingulate: β = –2.40×10⁻⁵, p = 0.294; right posterior cingulate: β = –1.50×10⁻⁵, p = 0.574). Brain volumes calculated as a ratio of intracranial volume. Shaded regions around the regression lines represent 95% confidence intervals of the model estimates, with individual points denoting participant data.


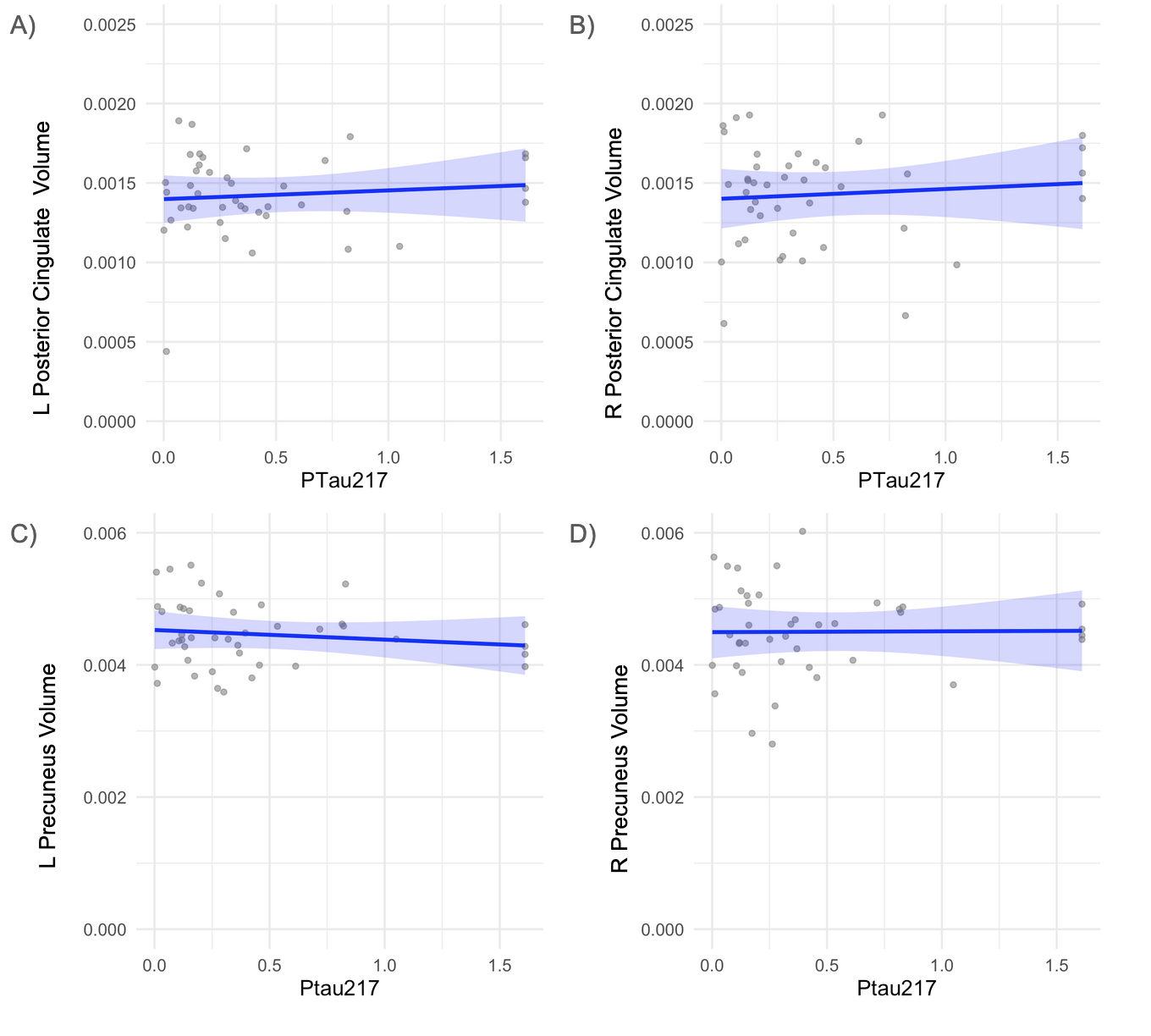


**Supplementary Figure 2.** Associations between plasma **pTau217** and regional brain volumes.Panels display linear regression models relating plasma pTau217 to (A) left precuneus, (B) right precuneus, (C) left posterior cingulate, and (D) right posterior cingulate volumes. No associations were statistically significant (left precuneus: β = –1.50×10⁻⁴, p = 0.422; right precuneus: β = 1.20×10⁻⁵, p = 0.960; left posterior cingulate: β = 5.50×10⁻⁵, p = 0.561; right posterior cingulate: β = 6.10×10⁻⁵, p = 0.604). Brain volumes calculated as a ratio of intracranial volume. Shaded gray regions show 95% confidence intervals around regression lines, with points representing individual participants.
